# Trial Reporting in Immuno-Oncology (TRIO): an American society of clinical oncology-society for immunotherapy of cancer statement

**DOI:** 10.1186/s40425-018-0426-7

**Published:** 2018-10-19

**Authors:** Apostolia M. Tsimberidou, Laura A. Levit, Richard L. Schilsky, Steven D. Averbuch, Daniel Chen, John M. Kirkwood, Lisa M. McShane, Elad Sharon, Kathryn F. Mileham, Michael A. Postow

**Affiliations:** 1 University of Texas MD Anderson Cancer Center, Houston, TX USA; 2 American Society of Clinical Oncology, 2318 Mill Rd, Alexandria, VA 22314 USA; 3 Bristol-Myers Squibb, Princeton, NJ USA; 4 Memorial Sloan Kettering Cancer Center and Weill Cornell Medical College, New York, NY USA; 5 Genentech, South San Francisco, CA USA; 6 University of Pittsburgh Cancer Institute, Pittsburgh, PA USA; 7 National Cancer Institute, Bethesda, MD USA; 8 Atrium Health, Charlotte, NC USA

## Abstract

**Purpose:**

To develop recommendations for clinical trial reporting that address the unique efficacy, toxicity, and combination and sequencing aspects of immuno-oncology (IO) treatments.

**Methods:**

ASCO and the Society for Immunotherapy of Cancer (SITC) convened a working group that consisted of practicing medical oncologists, immunologists, clinical researchers, biostatisticians, and representatives from industry and government to develop Trial Reporting in Immuno-Oncology (TRIO) recommendations. These recommendations are based on expert consensus, given that existing data to support evidence-based recommendations are limited.

**Conclusion:**

The TRIO recommendations are intended to improve the reporting of IO clinical trials and thus provide more complete evidence on the relative benefits and risks of an IO therapeutic approach. Given the rapid expansion of the number of IO clinical trials and ongoing improvements to the evidence base supporting the use of IO treatments in clinical care, these recommendations will likely need regular revision as the IO field develops.

## Introduction

Cancer immunotherapies are treatments in which the primary mechanism of action is the generation of an immune response against cancer. The US Food and Drug Administration has approved multiple cancer immunotherapies for treating different types of cancer. These cancer immunotherapies or immuno-oncology (IO) treatments include but are not limited to cytokines, interferons, and immune checkpoint blocking agents, such as anti-cytotoxic T-lymphocyte antigen 4 (CTLA-4; ipilimumab), anti-programmed cell death protein 1 (PD-1; nivolumab, pembrolizumab), and anti-programmed death-ligand 1 (PD-L1; atezolizumab, avelumab, durvalumab) agents and genetically modified T-cell therapies (axicabtagene ciloleucel, tisagenlecleucel). Several novel IO treatments are also being explored, such as modified cytokines, cell-based products, oncolytic viruses, CD3 bispecific antibodies, and various vaccine platforms. Clinical trials have been essential to advancing the use of immunotherapies in cancer treatment, and bio-medical journals have been a key mechanism for disseminating this knowledge.

To improve the quality of scientific publications, biomedical journals have adopted guidelines for reporting the design, conduct, analysis, and results of clinical trials (eg, the Consolidated Standards of Reporting Trials [CONSORT]), tumor marker studies (eg, Reporting Recommendations for Tumor Marker Prognostic Studies [REMARK]), and other study designs [1–3]. The principles from CONSORTand REMARK can be applied to IO trials. For example, when novel predictive and prognostic biomarkers are reported in IO trials, investigators should follow the REMARK guidelines, and they should indicate which hypotheses were predefined and which exploratory hypotheses are post hoc. IO therapies, however, use distinct mechanisms of action related to the generation of immune responses and may exhibit unique efficacy and toxicity effects compared with traditional cancer therapies such as chemotherapy. These features of IO therapies may lead to additional considerations for reporting the design, conduct, analysis, and results of IO clinical trials.

In fall 2016, ASCO and the Society for Immunotherapy of Cancer (SITC) convened a working group to develop clinical trial reporting recommendations that address the unique efficacy, toxicity, and combination and sequencing aspects of IO treatments. The working group consisted of practicing medical oncologists, immunologists, clinical researchers, biostatisticians, and representatives of industry and government. It met via a series of conference calls and held an in-person meeting in February 2017.

This ASCO-SITC statement presents 12 specific reporting recommendations—Trial Reporting in Immuno-Oncology (TRIO)— to improve the interpretation and comparison of efficacy and toxicity end points and the combination and sequencing of treatments in IO clinical trials (Table 1). These recommendations are based on expert consensus, given that existing data to support evidence based recommendations are limited. They are, in part, more prescriptive than other reporting guidelines because they recommend that certain analyses be performed and that results be presented to facilitate understanding and comparison across IO trials. As a result, the recommendations are practical, and hopefully they will lead to better trial conduct and relevant data collection. In addition, the recommendations will likely require regular updating as data from IO clinical trials emerge.

**Table 1:** Trial reporting in immuno-oncology (TRIO) standards

Reporting Standards	
Efficacy reporting standards	
1. Report the criteria used to evaluate response to therapy and the rationale for the chosen criteria.	
2. Include spider plots or swimmer plots in efficacy descriptions to better report kinetics of response (Figs 1 and 2).	
3. Report how disease control rate is defined and how its components are assessed.	
4. Report criteria that allow patients to continue treatment beyond disease progression.	
5. Report the number (proportion) of patients who are treated beyond progression, treatment beyond progression duration, emergence of new toxicity, and efficacy after initial progression.	
6. Report progression-free survival and overall survival using Kaplan-Meier analyses.	
Toxicity reporting standards	
7. Differentiate between the clinical diagnoses of IO toxicity and the specific symptoms that led to the diagnoses.	
8. If the prespecified clinical diagnoses used in data collection belong to categories such as “immune-related adverse events” or “adverse events of special interest,” report how these terms are defined and why these categories were selected for trial reporting	
9. Report all toxicity by specific grade.	
10. Report clinical interventions used to manage IO toxicity (Table 2).	
11. Report time of onset and duration of IO toxicity (Table 2).	
Combination or sequencing of immunotherapies reporting standard	
12. Report the scientific hypothesis for the combination or sequence on the basis of preclinical and/or clinical data as well as the rationale for the selection of the particular dose(s) and sequence of agents.	

Standards 1 to 5 and 7 to 11 are unique to immuno-oncology (IO) therapies

## Efficacy reporting standards for IO trials

Immunotherapies can exhibit unusual patterns of response because of the temporal aspects of the generation of immunity, the dynamic nature of the anticancer immune response, and the impact of tumor inflammation on tumor measurements. Specifically, there may be lag time before efficacy of IO therapies becomes evident, presumably because of the time required to activate an anticancer immune response. Once active, the anticancer immune response can fluctuate over time based on several host intrinsic and extrinsic factors [4]. It is also possible that tumor inflammation makes some tumors seem to be larger on imaging scans after the initial target measurement, which complicates response assessment [5]. To capture these atypical patterns of response, various immune-related response criteria have been proposed to define efficacy in IO clinical trials [5–9]. Because these criteria are not yet fully validated, the reporting recommendations in this section are intended to address the heterogeneous methods currently used to report efficacy across IO clinical trials and to ensure that investigators report the information necessary to enable objective assessment of antitumor efficacy.

### Defining antitumor activity

#### Report the criteria used to evaluate response to therapy and the rationale for the selected criteria

Response Evaluation Criteria in Solid Tumors (RECIST) are used to evaluate antitumor activity of oncology drugs in most clinical trials, including most IO trials [10]. These criteria were developed for trials of conventional chemotherapy in patients with solid tumors and declare progression when existing tumors increase in size by a certain amount (eg, ≥ 20% in tumor measurements) or if new tumors develop. Emerging evidence on IO therapies suggests that RECIST may underestimate the benefit of some IO approaches by declaring progression too soon in patients who ultimately benefit from treatment [11, 12]. Several iterations of IO-specific response criteria have been proposed in selected disease settings (ie, immune-related RC [irRC], irRECIST, iRECIST, imRECIST) [5–9]. These criteria have important differences, such as whether the tumor burden is calculated as the sum of the longest diameters (unidimensional) or the sum of the product of perpendicular diameters (bidimensional). None of the IO-specific response criteria have been uniformly adopted across all IO trials [13]. While they are being validated, investigators should specify which criteria were used, whether they were prespecified, and the rationale for their selection. To provide comparisons with prior clinical trials, consideration should be given to continuing to report responses according to standard RECIST criteria in parallel with the IO-specific response criteria.

#### Include spider plots or swimmer plots in efficacy descriptions to better report kinetics of response for nonrandomized trials

Most IO trials display efficacy as a waterfall plot. However, waterfall plots are inherently limited because only the patients’ best overall response is reported. They do not capture the temporal relationship of response after treatment is administered (ie, the timing of patients’ response and whether the response occurred during therapy or after discontinuation of therapy) nor do they provide any information about the duration of response. Thus, in addition to waterfall plots, investigators should report the kinetics of change in tumor burden using spider plots or swimmer plots to display more meaningful information about efficacy over time.

**Fig. 1:**
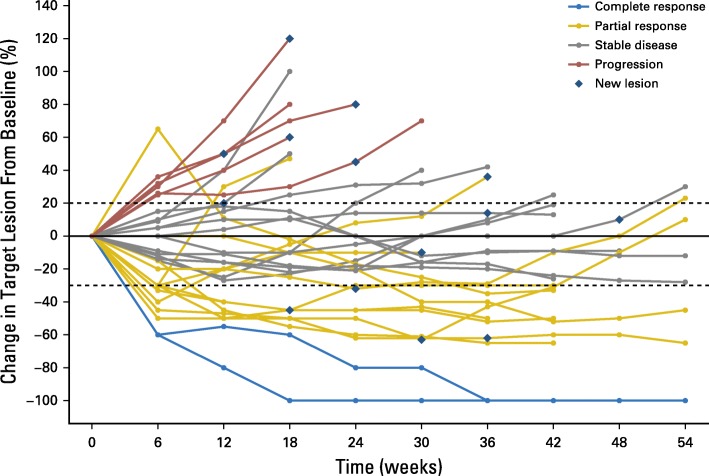
Hypothetical example of a spider plot showing tumor growth or shrinkage from baseline in a cohort of patients. Patients are often color coded to correspond to their best objective response. By displaying index lesion tumor burden over time, spider plots clearly illustrate tumor burden changes over time. They can demonstrate a favorable antitumor response in index lesions by showing their decrease, even in patients determined to have a best response of progressive disease as defined by the presence of a new lesion

**Fig. 2:**
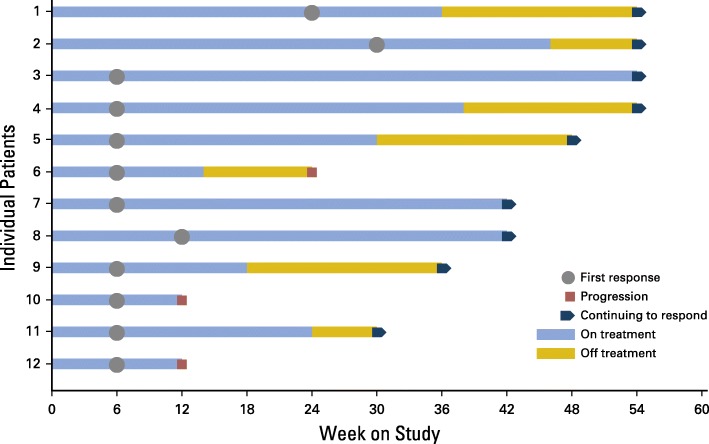
Hypothetical example of a swimmer plot showing time of objective response in relationship to duration of treatment and time of treatment cessation. Symbols along each bar and at the end of each bar could be used to represent various relevant clinical events, such as disease progression or start of a new anticancer therapy. Swimmer plots provide useful information about responses, which may start after cessation of immunotherapy, and about the potential persistence of these responses even without ongoing treatment. Continuation of response despite immunotherapy discontinuation is an important efficacy metric

Spider plots (Fig. 1) typically illustrate change in tumor burden over time within individual patients and usually include symbols indicating when new lesions appear. The major limitations of spider plots are that their interpretation is difficult when the number of patients is large and the representation of patients with progressive disease versus those who have complete response plus partial response plus stable disease is imbalanced. Specifically, the loss of data from the patients who have progressive disease may lead to misrepresentation of benefit for the overall population [14]. Therefore, they are most useful in reporting smaller trials or selected populations of interest within larger trials. Swimmer plots (Fig 2) typically depict the treatment course of individual patients and may show how long patients receive treatment, time off treatment, and duration of response or other relevant clinical events such as time of disease progression [15].

#### Report how disease control rate is defined and how its components are assessed

Investigators often collect and report nonstandardized end points in clinical trials using terms such as “disease control” and “clinical benefit.” These terms may include patients with objective response (complete and partial) and patients with stable disease, usually for a prespecified minimum number of months (eg, 6 months). When such composite end points are reported, they should be clearly defined, and the frequency and method of assessment of their components should be clearly described. For time-to-event end points, investigators should provide a clear definition of baseline (time 0) as well as what occurrences are considered censored and what are considered events.

Although the importance of clearly defining time-to-event end points is not unique to IO trials, it is especially relevant in the current IO trial landscape because of the vast number of nonrandomized IO trials investigating new agents alone and in various combinations. The controversial issue surrounds patients who are determined to have stable disease by standard imaging criteria. For example, a patient with stable disease for 6 months on an experimental IO therapy may have met a trial’s clinical benefit or disease control designation, but if that patient had a cancer with an indolent underlying disease biology, there may have been no significant drug benefit from the intervention. Nonrandomized studies are inherently limited in demonstrating true clinical benefit or disease control of an experimental approach because of the absence of a comparator arm, which is necessary to interpret time-to-event end points.

### Describing clinical events after disease progression

#### Report criteria that allow patients to continue treatment beyond disease progression

Historically, cancer clinical trials have required cessation of experimental therapy upon determination of disease progression. IO clinical trials, however, often allow patients to continue therapy beyond objective determination of progressive disease because of the recognition of a phenomenon known as pseudo-progression. Pseudo-progression is an event that can occur with IO therapies that denotes the appearance of new lesions (usually with shrinkage of baseline index tumor burden) or an initial increase in index lesion(s) with subsequent index lesion response by clinical or radiographic assessment [5, 7, 11, 12, 16].

Common criteria for treating beyond progression are that patients have stable or improved clinical condition, no severe laboratory abnormalities or adverse events associated with IO therapy, and lack of clinically significant additional progression on confirmatory subsequent imaging. However, there are no universally accepted criteria for defining what may happen in an IO clinical trial after disease progression. Thus, the specific criteria used in a trial to permit treatment beyond progression should be reported. This information should include the timing of the required confirmatory imaging. Investigators should also report whether alternative treatments were discussed with the patients and whether patients signed a consent form at the time of initial documentation of disease progression.

#### Report the number (proportion) of patients who are treated beyond progression and the treatment beyond progression duration, emergence of new toxicity, and efficacy after initial progression

It is critical to distinguish patients with pseudo-progression from true progression to avoid exposing the latter group to ineffective treatment that may be associated with adverse events [17]. It is also important to capture the incidence of pseudo-progression [18]. Therefore, investigators should report the overall number of patients who are treated beyond progression and their outcomes. Specifically, this information should include the number of patients treated beyond progression who experienced tumor response but ultimately had confirmed progression on a subsequent scan, the median duration of time patients were treated beyond initial progression before treatment was discontinued as a result of progression; and new toxicities that arose or existing toxicities that worsened while patients were treated beyond initial progression.

### Reporting progression-free survival and overall survival

#### Report progression-free survival and overall survival using Kaplan-Meier analyses

Investigators should report progression free survival and overall survival using Kaplan-Meier analyses in IO trials. The landmark methodology may be used for the correlation of progression-free survival or overall survival with response status [19]. By using this method to evaluate outcomes, patients who die early do not prejudicially influence the analysis of a postdiagnosis end point. Two noticeable aspects of the Kaplan Meier survival curves in IO trials are the presence of delayed separation of curves resulting from delayed clinical effect in the IO arm and presence of nonzero tail probabilities because of a higher proportion of patients experiencing sustained tumor control or prolonged survival in the IO arm. These two aspects of the survival curves could potentially lead to underestimation of trial duration and loss of power to detect treatment effects [20]. Any statistical test used to compare survival curves between treatment arms should be described in detail; the unique characteristics of IO Kaplan-Meier curves have led to varied recommendations concerning tests that differently weight survival differences early versus late in the time course [21, 22].

Investigators should also report whether patients were censored atthe time they started a new therapy. The date of earliest evidence of disease progression should be reported as the date of disease progression for patients who continued treatment beyond progression but were confirmed to have true progression on a subsequent assessment.

### Toxicity reporting standards for IO trials

Toxicities that patients experience from IO therapies (IO toxicity) differ from those with other cancer therapies, such as chemotherapy and molecularly targeted therapy. Immune check-point blockade, for example, is associated with organ-specific adverse events, such as hepatitis, colitis, dermatitis, pneumonitis, and endocrinopathies such as hypophysitis [23]. The adverse events of IO therapies can last longer or be more transient than adverse events from other treatment modalities. There is also variability in the onset of toxicity; for example, IO toxicity may begin after treatment cessation [24, 25]. Managing these toxicities requires interventions unique to the immune-based mechanism of action for IO therapies, including those related to chimeric antigen receptor T-cell therapies [26–28]. Nonetheless, most toxicities from immunotherapies are reversible with immune suppression that uses corticosteroids and/or other immunosuppressive agents such as mycophenolate mofetil and tumor necrosis factor-alpha antagonists (eg, infliximab) [29].

Despite the critical importance of knowing the safety profiles of cancer treatments, there is evidence that toxicity reporting is suboptimal in clinical trials [30–33], including IO trials [34]. Accurate and timely toxicity reporting in IO trials would provide critical details about the occurrence and management of IO toxicities. Thus, the reporting recommendations in this section are intended to ensure that investigators describe all captured toxicities, especially those unique to IO therapies, to enable better characterization of their safety profile. Some of the recommendations may require collecting information that is not standard in all clinical trials. However, the hope is that proposing these recommendations will inspire improved methods of collecting data in future clinical trials.

#### Differentiate between the clinical diagnoses of IO toxicity and the specific symptoms that led to the diagnoses

Clinical diagnoses of IO toxicity often describe a constellation of specific signs and symptoms that afflict a patient. For example, a patient receiving immunotherapy may have individual symptoms such as headache and fatigue. The clinical diagnosis for these symptoms, however, may be immunotherapy-related hypophysitis. Similarly, a patient who reports abdominal pain and diarrhea may ultimately be diagnosed with colitis. Both symptoms and diagnoses are reported in clinical trials, often describing the same event and resulting in confusion regarding the precise incidence of particular toxicities [35]. To avoid this confusion, investigators should report the clinical diagnoses of IO toxicity separately from the raw data of associated symptoms. They should also report the clinical evaluations that were used to determine a diagnosis of IO toxicity. The National Cancer Institute’s (NCI’s) Common Terminology Criteria for Adverse Events (CTCAE) is the current standard for toxicity reporting in clinical trials. To better reflect clinical diagnoses of IO toxicities, such as hypophysitis, the NCI recently updated to CTCAE v5.0 to clarify the symptoms and data required for the diagnosis of an IO toxicity [36]. Future updates will likely be necessary as knowledge of IO toxicities increases.

#### If the prespecified clinical diagnoses used in data collection belong to categories such as immune-related adverse events or adverse events of special interest, report how these terms are defined and why these categories were selected for trial reporting

The terms “immune-related adverse events” (irAE) or “adverse events of special interest” (AEOSI) are often used to describe clinical diagnoses of IO toxicity (eg, hypophysitis, colitis, hepatitis). Many clinical trials distinguish adverse events belonging to these categories from other toxicities and separately report them. When investigators use predetermined categories of toxicities, such as irAE or AEOSI, they should explain how and why certain toxicities were selected for inclusion within these categories and what criteria were met to attribute a toxicity to one of these terms [37].

#### Report all toxicity by specific grade

Clinical management of IO therapies would be better informed by increased granularity of toxicity grading data. Investigators usually report all grade toxicity and grade 3 to 4 toxicity separately to highlight the incidence of serious (grade 3 to 4) toxicities relative to the total number of toxicities observed. Given the nature of IO toxicities, investigators should report the specific grade of each toxicity collected in a clinical trial. There are clinically meaningful differences between grade 1 and 2 toxicities as well as between grade 3 and 4 toxicities. For example, grade 1 pneumonitis is defined in the CTCAE as asymptomatic and based on clinical or diagnostic observations, whereas grade 2 pneumonitis indicates symptoms interfering with instrumental activities of daily living for which medical intervention is indicated. Grade 3 pneumonitis is associated with oxygen initiation, whereas grade 4 toxicity is life threatening and accompanied by intubation. Grade 2 or higher immune-related pneumonitis usually requires that IO treatment be withheld and that steroids be initiated. When only grade 3 to 4 toxicity is reported in IO trials, grade 2 events are not clearly captured despite being clinically relevant. Reporting toxicity by each specific grade (ie, separate columns in tables for grades 1 to 4 adverse events) will optimize the value of the available information on the toxicity profile of IO therapies.

#### Report clinical interventions used to manage IO toxicity

Table 2 illustrates a proposed format for summarizing the clinical consequences and management of adverse events (ie, dose delays, use of immunosuppression) in IO trials, providing a comprehensive description of the toxicity profile not otherwise captured by standard CTCAE criteria. Several retrospective studies have reanalyzed immunotherapy toxicity data from clinical trials with the goal of capturing the clinical management associated with immunotherapy toxicity, including the use of various forms of immunosuppression and rates of emergency room visits and hospital admissions for toxicity management [38, 39]. However, investigators should ideally report this information for at least the most common toxicities in the initial publications of prospective IO clinical trials. Investigators should also comment in the discussion section of manuscripts on whether the reported toxicity management in the clinical trial adhered to consensus management guidelines, at least for unexpected toxicities or those that significantly affected the patients. Professional organizations such as the National Comprehensive Cancer Network, SITC, and ASCO have published guidelines for generally managing IO toxicities [26, 27]. There are also specific toxicity management guidelines for chimeric antigen receptor T-cell and other adoptive transfer cell therapies, given their highly unusual toxicities [28].

**Table 2:** Reporting of clinical consequences of toxicity

Patients Who Experience Toxicity									
Adverse Event	Dose Delay (No. and Proportion of patients	Dose Discontinuation^a^ No. and proportion patients)	Timing of Toxicity Onset (median and range)^b^	Use of High-Dose^c^ Steroids (No. and proportion of patients	Duration of High-Dose Steroid Use (median and range	Duration of Dose Tapering^d^ (median and range)	Additional Immune- Suppressing Agents (No. and proportion of patients who required escalation beyond steroids; specify drugs)	Time to Resolution of Toxicity^e^ (median and range, percent of patients with unresolved toxicity)	Emergency Center Visit/ Hospitalization (No. and proportion of patients)
Adverse event 1									
(e.g., colitis)									
Adverse event 2									
Adverse event 3									
Adverse event 4									

^a^Defined as the inability to continue on the protocol; may include irreversible toxicity and toxicity resulting in ineligibility for subsequent treatment

^b^Days from cycle 1, day 1 to time of onset (include cycle, day and period from initiation of treatment)

^c^Defined as at least 40 mg prednisone equivalents per day

^d^If the protocol required collecting this information

^e^Define specifically if “resolution” refers to return to grade 1 or 0 (indicate whether this includes patients who are on steroids to manage adverse events)

#### Report time of onset and duration of IO toxicity

In addition to capturing the clinical management of adverse events resulting from IO therapies, Table 2 also captures the timing of toxicity onset and of toxicity resolution, which enables determination of duration of toxicity. Because IO toxicity can be long lasting [24, 25], reporting the duration of toxicity is arguably as clinically important as severity (grade) of toxicity. These data would aid clinical management and provide information that is useful to the design and interpretation of subsequent combinatorial IO trials. For example, if drug X as a single agent is not known to cause transaminitis and drug Y is not known to cause transaminitis until, on average, 6 weeks after treatment initiation, significant transaminitis occurring within 2 weeks of therapy in a combination study of drug X plus drug Y could raise the possibility of synergistic toxicity. To clearly illustrate this information, investigators could enhance swimmer plots to capture time of toxicity onset and duration of toxicity relative to when a therapy was given or develop new plots with greater informational content [40].

### Combination or sequencing of immunotherapies reporting standard

An increasing number of clinical trials are testing immunotherapies in binary, ternary, and more complex combinations and sequences. These studies are evaluating IO therapies with each other and with targeted therapies, chemotherapy, and radiation therapy [41]. There are already several Food and Drug Administration–approved IO combination treatments [42]. The rationale for conducting combination and sequencing trials is that the use of multiple treatments may broaden and/or deepen the antitumor activity of IO drugs. The combination of nivolumab and ipilimumab, for instance, has a 58% response rate in patients with metastatic melanoma, which is greater than the response rate of either therapy alone [43, 44]. However, combining therapies also increases the riskof added or novel toxicity. The rate of grade 3 to 4 toxicity in patients receiving nivolumab plus ipilimumab is close to 60%, which is higher than that in patients receiving either drug as a single agent [43–45]. The goal of the reporting standard in this section is to ensure that the justification for combining or sequencing treatments in IO trials is adequately reported, including biologic and/or clinical evidence for contribution of the individual components.

#### Report the scientific hypothesis for the combination or sequence, as well as the rationale for the selection of the particular dose(s) and sequence of agents

Investigators should report preclinical and/or clinical data that support the hypothesis that a combination or sequencing of therapies in an IO trial is likely to have additive or synergistic antitumor activity and a favorable therapeutic index [17]. They should also provide a rationale for the selection of the particular dose and sequence of agents. This information can be illustrated in a figure that captures the dose and sequence of the agents administered, dose level, administration frequency, length of dosing interruption, duration of treatment of each agent, and whether the treatments are administered sequentially or concurrently. Many IO therapies have only a modest dose-response relationship and have the potential for prolonged biologic effects after discontinuation of treatment. Determining the dose for IO combinations is further complicated by the fact that nonstandard doses or schedules of the individual agents may be necessary. Some IO therapies may have limited benefit when used alone but high potential for benefit when used in combination [46].

In conclusion, the 12 reporting recommendations presented in this statement are intended to improve the reporting of IO clinical trials and thus provide more complete evidence on the relative benefits and risks of an IO therapeutic approach. Although some of these recommendations may be relevant for other types of cancer clinical trials, the goal of these recommendations is to address the unique efficacy, toxicity, and combination and sequencing characteristics of IO treatments. Given the rapid expansion of the number of IO clinical trials and ongoing improvements to the evidence base that supports the use of IO treatments in clinical care, these recommendations will likely need regular revision as the IO field develops. This iterative process will be critical to ensuring that IO clinical trial data are interpreted appropriately and are ultimately useful to improving the care of patients with cancer.
